# The Jackson Laboratory Mouse Genome Informatics Site: Version 2.3.2

**DOI:** 10.1002/1097-0061(20000630)17:2<134::AID-YEA19>3.3.CO;2-T

**Published:** 2000

**Authors:** Jo Wixon

**Affiliations:** School of Biological Sciences University of Manchester Manchester M13 9PT UK


**Yeast**
*Yeast* 2000; **17**: 134–145.

Website Review

### The Jackson Laboratory Mouse Genome Informatics Site: Version 2.3.2

http://www.informatics.jax.org/

**Site authors: Mouse Genome Informatics, The Jackson Laboratory, Bar Harbor, Maine. Project PI: Janan Eppig. All screen views from the website are reproduced with the kind permission of Janan Eppig.**

Jo Wixon
*Managing Editor—School of Biological Sciences, University of Manchester, Manchester, M13 9PT, UK*

### Site structure

The main page is broken down into three sections:

**Searches, data and reports** (searches of subsections of the databases):
- Genetic and phenotypic data.
- Mapping and marker data.
- Strains and polymorphisms.
- Expression data (some entries include figures of results from published work).
- Mammalian homology data (includes Oxford grids and comparative maps).
- Database reports (view the data as lists, as an alternative to searching).
- Chromosome committee reports.
- Contributed datasets (these have not been integrated into the databases).

**Prototypes** (services and databases that are still under development):
- BLAST service.
- Mouse Tumour Biology database (MTB; Bult *et al.*, [2000]).
- Rat genome data.

**Other MGI resources**:
- Advice on nomenclature.
- Data submission guidelines.
- User support page.
- E-mail-based discussion lists (users can subscribe to these).

### Site guide

The main page of Mouse Genome Informatics (MGI) is a list of links to subpages (Figure 1). This topic-based breakdown of the data helps the user to find the right page to answer his/her query and I have followed this structure for the site guide. Most of the links go through to a page with a list of services, headed by the same title. Each title forms a link to the relevant section of the site overview; this gives information about the available data, and an explanation of what the searches offered on the page are designed to do. In some cases, the titles link to instructions on how to use the searches and tools. Above these titles are links to each of the other search topic pages; these allow more rapid movement around the site. Between these links and the title, on every page, is a pull-down menu, for those who wish to go directly to a search form. This includes a link to the 'quick [search] forms', which have fewer fields to be completed.

### Searches, data and reports

This section provides searches of subsections of the vast collection of mouse data held in the Mouse Genome Database (MGD; Blake *et al.*, [1999]), the Mouse Genome Sequence Database (MGS) and the Gene Expression Database (GXD; Ringwald *et al.*, [1999]) at the Jackson Laboratory. In addition to the links described below, there is a small query box for a quick search of the genes catalogue.

### Genetic and phenotypic data

**Searches**

The 'Genes, Markers and Phenotypes' link leads to a query form that allows the user to search by locus symbol, name, map position, and many other characteristics. Clicking on 'Combined Mouse/Human Phenotypes' leads to a form for a simulta-

Copyright © 2000 John Wiley & Sons, Ltd.

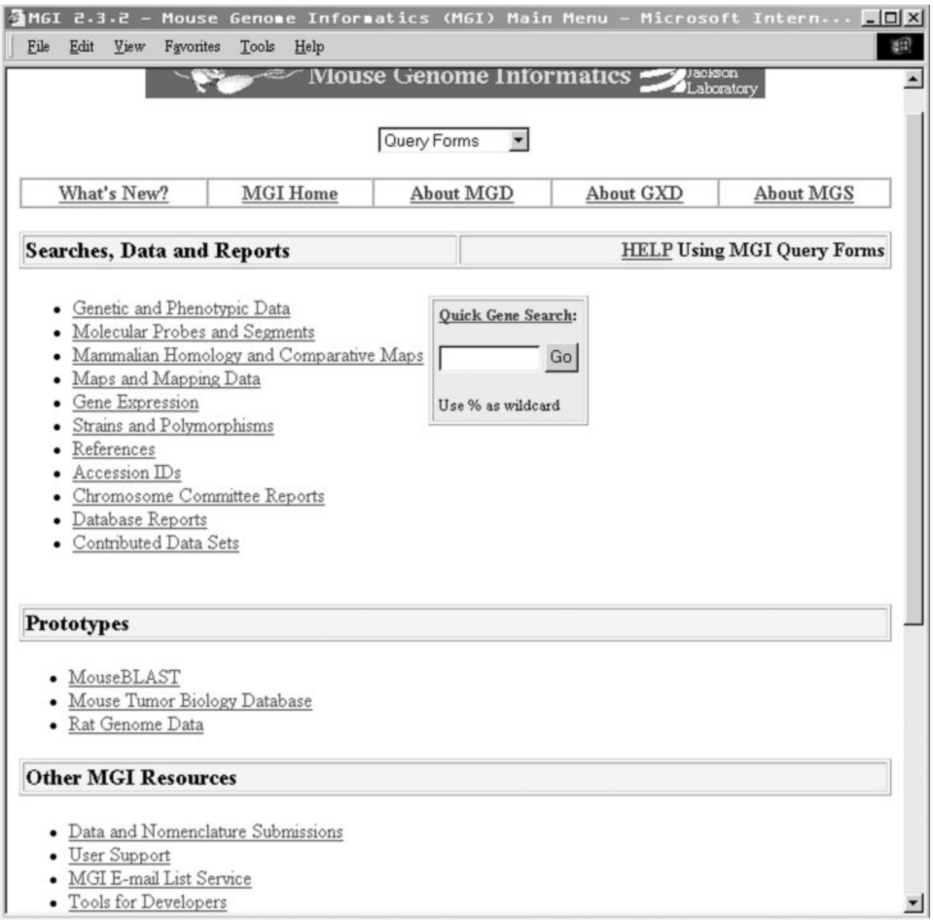

**Figure 1.** The main page of the Mouse Genome Informatics website neous keyword search of the Mouse Locus Catalogue (MLC) and Online Mendelian Inheritance in Man (OMIM). Users can also search for polymorphisms by type (PCR or RFLP), locus symbol, map position or strain. A search for the characteristics of inbred strains in M. Festing's Listing of Inbred Strains of Mice and Rats is also offered. The 'Quick Forms' link 'does exactly what it says on the tin' and leads to a broken down listing of all possible searches, each of which leads through to a simple form with fewer criteria, allowing a more rapid search.

#### Reports

The 'Chromosome Committee Reports' link leads to an archive of the reports of each chromosome committee, for the current year and previous years going back to 1995. The 'database reports' link leads to MGI data on genetic markers, mammalian homology, polymorphisms, and gene expression patterns. There are also some general statistics and links to nomenclature information. The markers are sorted by name or chromosome and the last month's updates are available. The mouse–human homology and mouse–rat homology results are sorted by chromosome or gene symbol in each organism.

#### Data submission tools and guidelines

The 'Mouse Nomenclature Guidelines and Locus Symbol Registry' link is intended to be a guide for users who wish to name and register a gene or locus. It gives details of the nomenclature committee and their recommendations and gives links to databases so that users can check that their chosen name is not already in use. The second link, 'Submitting Mouse Genome Data', is very self-explanatory. It contains guidelines for those who

Copyright © 2000 John Wiley & Sons, Ltd.

wish to submit mouse data to MGD. There are detailed instructions and examples of each section of the forms.

### Molecular probes and segments

### Searches

This page links to a search of all the information held on probes and segments in the mouse maps. This search can yield information about the source of a probe, to which loci it hybridized and the sequence of the probe if known. There is also a link to the 'Quick [query] forms'.

### Reports

There is a link to the probes section of the 'database reports' pages described in the 'Genetic and Phenotypic Data' section.

### Mammalian homology and comparative maps

This page has links to services which access homology information for mouse, human and 17 other mammalian species, most of which has been extracted from published scientific literature. In particular, the Mammalian Homology search and the Oxford grid tool will be of great interest to any readers working on mammalian genomes. Since there is no obligation to use the mouse as one of the species, any pair from the 17 species can be compared.

### Searches

The 'Mammalian Homology' search offers several options, from obtaining all the homologous genes between two species, through specifying a chromosome or chromosome region, to specifying the name of a particular marker (gene) or the author of a paper. All of the titles of the search criteria in the form link to an explanation of how to enter a search term.

The 'Oxford grid' tool will produce a plot of the homologous loci between two genomes from a choice of seven. The $x$ and $y$ axes of the plot are the chromosomes of the two species, such that there is a cell for each possible combination of chromosomes (Figure 2). The number of matches for that pair of chromosomes appears in the box and colour coding is given to aid the user to spot chromosomes with many genes that are homologues. Clicking on a box from the grid leads to a table of all the homologous loci on that pair chromosomes. If mouse is one of the chosen organisms, then each chromosome number on the mouse axis links to the entire comparative map of that mouse chromosome with the second chosen organism. Another service offered in this section of the site is to build a 'comparative map'. The user can specify the format of the map, the source of information for the map and the chromosome or region of interest in the mouse genome. Next, the types of markers to be included in the map are selected, and the second genome is chosen from a list. There is even an option to add a marker/s that the user has mapped independently. Once the form has been completed, the map is obtained by clicking the 'retrieve' button; an example of a short-range map is shown in Figure 3. Whilst this makes an excellent first port of call for researchers wanting to identify regions of synteny between the mouse genome and another mammalian genome, it does only provide coarse data, i.e. mapped orthologues, rather than taking advantage of the sequencing data which is available.

This section also contains a link to the quick forms, described previously.

### Reports

The 'Mouse/*Drosophila* Homologies' link leads to a table of homologous genes found in both the mouse and fly genomes, compiled by Michael Ashburner. There is also a link to the Mammalian Homology section of the database reports page.

### Homology criteria

There are links to the report of the 1995 HUGO (Human Genome Organization) committee on comparative mapping and to the homology criteria (as defined by this committee) that are used by MGD to describe mammalian homologues.

### Maps and mapping data

The title of this page links to detailed guidelines for using the searches offered on the page.

### Searches

The 'Mapping Data' search allows users to retrieve mapping data on any chosen marker or markers. The type of experiment used can be specified, e.g. FISH, or all of the data available can be retrieved. Other criteria can be used for this search, such as chromosome and experiment type, to yield all of the mapping data produced for chromosome 11 using somatic cell hybrids (HYBRID), for example. Other

Copyright © 2000 John Wiley & Sons, Ltd.

Total Homologies: 4207

Total mapped in both species: 3031

mouse, laboratory (columns); human (rows)

| | 1 | 2 | 3 | 4 | 5 | 6 | 7 | 8 | 9 | 10 | 11 | 12 | 13 | 14 | 15 | 16 | 17 | 18 | 19 | X | Y | XY | UN | MT | |
|---|---|---|---|---|---|---|---|---|---|---|---|---|---|---|---|---|---|---|---|---|---|---|---|---|---|
| 1 | 94 | 1 | 84 | 93 | 2 | | | 5 | | | 1 | 1 | 6 | | | | | | | | | | 82 | | 1 |
| 2 | 74 | 44 | | | 6 | 27 | | | | | 8 | 17 | | | 1 | | 9 | 1 | | | | | 45 | | 2 |
| 3 | 1 | | 18 | 1 | | 27 | | 1 | 54 | | | | | 10 | | 30 | 1 | | | | | | 48 | | 3 |
| 4 | | | 24 | | 72 | 4 | | 19 | | | | | 2 | | | | | | 1 | | | | 24 | | 4 |
| 5 | 1 | | | | | | | | | | 44 | | 51 | | 12 | | 4 | 26 | | | | | 33 | | 5 |
| 6 | 5 | | | 5 | 1 | | 1 | | 7 | 29 | | | 18 | | | | 106 | | | | | | 30 | | 6 |
| 7 | | 1 | | | 62 | 61 | 1 | | | | 13 | 13 | 5 | | | | 1 | | | | | | 23 | | 7 |
| 8 | 6 | | 6 | 10 | | | | 24 | | | | | | 17 | 30 | 3 | | | | 1 | | | 23 | | 8 |
| 9 | | 41 | | 50 | | | | | | | | | 8 | | | | | | 11 | | | | 25 | | 9 |
| 10 | | 11 | | | | 3 | 12 | | | 12 | | | 1 | 16 | | | | 3 | 46 | | | | 18 | | 10 |
| 11 | | 32 | | 1 | | | 70 | | 45 | | 1 | | | | | | | | 43 | | | | 53 | | 11 |
| 12 | | 1 | 1 | | 20 | 63 | 1 | | | 41 | | | | | 44 | | | | | | | | 39 | | 12 |
| 13 | 2 | | 2 | | 8 | | | 12 | | | | | | 20 | | | | | | | | | 12 | | 13 |
| 14 | | | 1 | | | | 1 | | 1 | 1 | | 43 | | 26 | | | | | | 1 | | | 26 | | 14 |
| 15 | | 24 | | | | | 32 | | 31 | | 1 | | 1 | | | | | | | | | | 15 | | 15 |
| 16 | | 1 | | | | | 20 | 48 | | | 8 | | | | 1 | 15 | 15 | | | | | | 23 | | 16 |
| 17 | | | 1 | 1 | | 1 | | 1 | | | 186 | 2 | | 1 | | | | | | | | | 43 | | 17 |
| 18 | 3 | | | | 2 | | | | | | | | | | | 1 | 4 | 31 | | | | | 8 | | 18 |
| 19 | | | | | | 1 | 92 | 26 | 10 | 26 | 1 | 1 | | | | | 15 | | | | | | 49 | | 19 |
| 20 | | 76 | | | | | | | | | | | | | | | | | | | | | 11 | | 20 |
| 21 | | | | | | | | | | 22 | | | | | | 27 | 6 | | | | | | 13 | | 21 |
| 22 | 1 | | | | 6 | 2 | | 3 | | 10 | 8 | | | | 35 | 27 | | | | | | | 13 | | 22 |
| X | | | | | | | | | | | 2 | | | 1 | | | | | 1 | 160 | | | 22 | | X |
| Y | | | | | | | | | | | | | | | | | | | | | 6 | | | | Y |
| XY | | | | | | | | | | | | | | | | | | | | | | | | | XY |
| UN | 9 | 18 | 11 | 12 | 9 | 3 | 12 | 7 | 7 | 14 | 13 | 6 | 9 | 1 | 6 | 5 | 10 | 3 | 5 | 4 | | | 334 | | UN |
| MT | | | | | | | | | | | | | | | | | | | | | | | | 37 | MT |

**Figure 2.** An Oxford Grid comparing the mouse and human genomes. The chromosomes from the two species form the axes of the plot, and the numbers in each cell indicate how many homologues there are on each pair of chromosomes. The cells are colour coded to give a quick indication of the number of homologues. Grey = 1 Blue = 2–10 Green = 11–25 Orange = 26–50 Yellow = 50 +. This grid represents 4232 homologies, 3040 of which have been mapped in both species fields include author name and MEDLINE Accession No. for a reference, this will help users who have read a paper and want to see the mapping data associated with it. The results are presented as a table, with a brief description and links to the data and to the relevant publication if there is one.

The 'recombinant inbreds strain distribution patterns' page offers users a choice of recombinant inbred strains and chromosomes. This tool retrieves a table showing the distribution of marker alleles in a selection of mice from the chosen strain. Letters are used to indicate which parental allele of each marker has been inherited by each of the progeny. This allows users to determine where recombinations have occurred in that chromosome, for each of the progeny. The table has links to the experiments used to determine the status of each allele and the related publications. Each marker on the left of the table forms a link to the full marker record. A similar search of recombinant congenic strain data is also provided.

The 'Mapping Panel' tool offers a selection of mapping panel strains and a chromosome selector. The table retrieved is similar to the inbred and congenic strain tables. It shows the allele distributions for all of the markers on that chromosome in a large panel of progeny from the selected cross.

This page also offers tools to build graphical linkage, cytogenetic or physical maps. The linkage map tool is the same tool as the one used to build a

Copyright © 2000 John Wiley & Sons, Ltd.

comparative map, the output looks the same and can also have comparative data shown on it. The cytogenetic map link leads to a page on which you select the chromosome whose cytogenetic map you wish to see.

The physical map link leads to a table of the integrated linkage and physical maps produced by the Whitehead Institute and MIT Center for Genome Research (CGR). These are broken down by chromosome (Figure 4) and into singly- or doubly-linked contigs. There is also a link to the quick query forms.

#### Reports

This has a link to the chromosome committee reports, since these contain mapping data.

#### Radiation hybrid mapping resources

This page also has links to the Jackson lab, EBI and Whitehead Institute/MIT CGR Mouse Radiation Hybrid databases. The Jackson lab and EBI resources are independent databases that are separately maintained; however, the curators of these two databases are collaborating, and send regular updates to each other.

#### Software tools

Links to related tools are available from this page; the 'Encyclopedia of the Mouse Genome' and Map Manager. The Encyclopedia tool allows for graphical representation of downloaded linkage maps and Map Manager is a Macintosh compatible database for analysis of genetic mapping data.

### Gene expression

The title link on this page leads to an in-depth explanation of GXD, including information on who is contributing and an explanation of the concept behind the project. GXD is the result of a collaboration between the Jackson Laboratory and the UK MRC Human Genetics Unit in Edinburgh (**http://genex.hgu.mrc.ac.uk/.**)

#### Searches

There are three searches offered on this page. The first is a search of the GXD index, which is a collection of references relating to gene expression data. The index can be searched with several types of terms, including a gene name, an author name, journal information or assay type.

The form for querying the gene expression data allows the user to limit the search to a specified gene, chromosome or region of a chromosome. There are also options to specify the developmental stage or anatomical structure observed and the method used for detection of expression. The search retrieves a list of the expression data pertaining to the specified gene or region (Figure 5). Each gene in the list links to further data on the gene, and the reference code leads through to the relevant publication. The assay codes link through to tables that describe the raw data, and images (often extracted from publications), where possible. This service is particularly impressive, since several databases do not seem to supply expression pattern images at the moment, despite the fact that this type of data is arguably best interpreted visually.

The third search is a cDNA clone query form. This allows users to ascertain whether there is a cDNA clone available for a given gene or for all the genes on a chromosome or in a chromosomal region of interest. Since the origin of a cDNA clone can be used to infer expression information, there are also several fields for describing the origin of a cDNA clone. Using these, users can ask questions like, 'is there any cDNA clone evidence for expression of my favourite gene in brain?'

#### Reports

A link to the database reports is also available here because the reports contain information on the origin of cDNA libraries and on genes that undergo alternative splicing.

### Strains and polymorphisms

#### Searches

The 'Polymorphisms' search and 'Inbred Strain Characteristics' search offered here are the same as those available from the 'Genetic and Phenotypic Data' page.

#### Nomenclature for strains

The 'Strain Nomenclature Guidelines' link jumps straight to the section on strain nomenclature of the Mouse Nomenclature Rules and Guidelines. There is also a link to approved nomenclature for strain 129 and substrains.

#### Other contributed data sets

The 'Polymorphisms in Microsatellite Markers for 129/J vs. Other Inbred Strains' link leads to a list of externally contributed datasets that have not been

Copyright © 2000 John Wiley & Sons, Ltd.

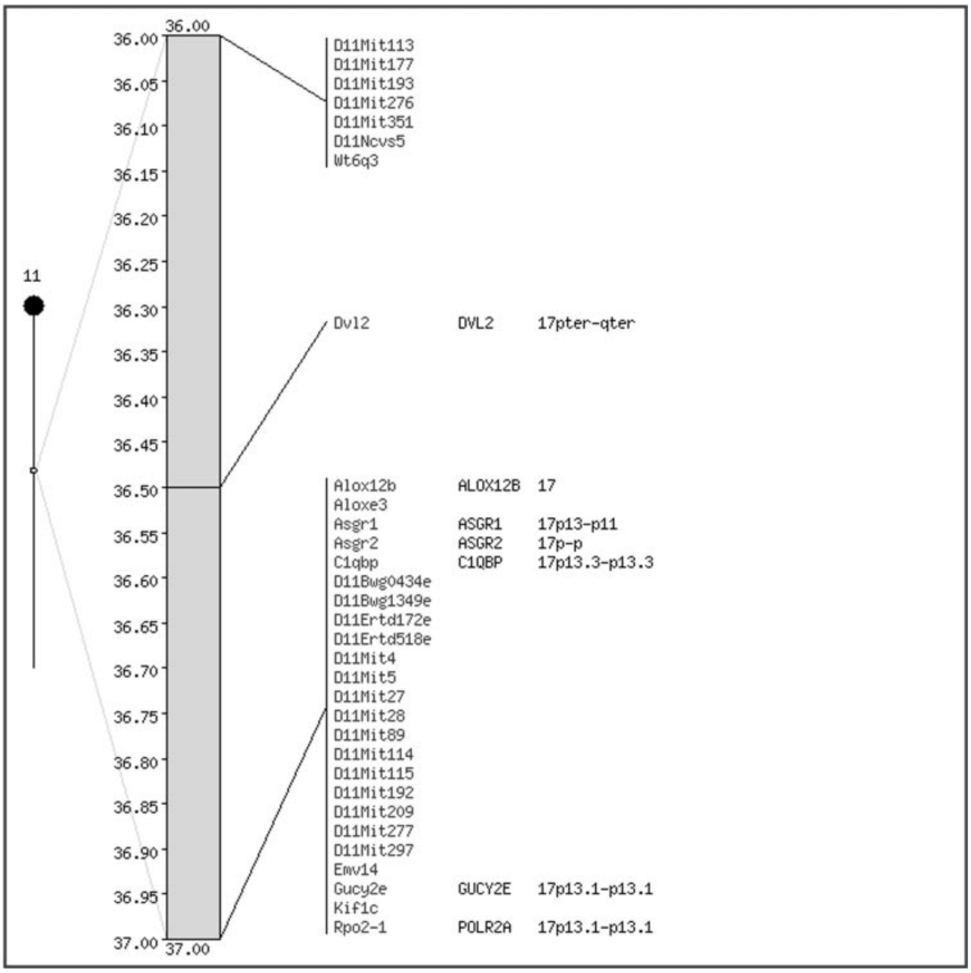

**Figure 3.** A comparative map made using the MGD map of mouse chromosome 11 from 36–37 cM against the human genome map. This region is part of the syntenic region that is indicated by the yellow coded cell for the comparison between mouse chromosome 11 and human chromosome 17 in the Oxford grid in Figure 2. This map includes DNA segments and genes of any category as markers. The mouse markers are listed in the left hand column, each one links to a full record on that marker. The human markers, and the chromosomal locations to which they have been assigned, are listed in the right hand column incorporated into the database. All of the datasets in this section relate to mapping, reporting on allele distributions in various strains, or the use of SSLP markers to place ESTs on to the map. The 'Genealogy Chart of Inbred Strains' is a chart of the origins and relationships between the many inbred strains of mice that have been produced.

### References

This section offers a search of the entire collection of references that are held by MGI and a search of the GXD index, which is the same as the one offered on the 'Gene Expression' page. The reference search can be done using a MEDLINE Accession No., an author name, journal name or several other query terms. There is also a link to the quick forms, since there is a simpler search form for reference searching.

### Accession Ids

This page offers a search of the entire dataset for any records associated with that ID. For example, if a user knows the MGI ID for his/her favourite gene, using this search will retrieve reports involving

Copyright © 2000 John Wiley & Sons, Ltd. 

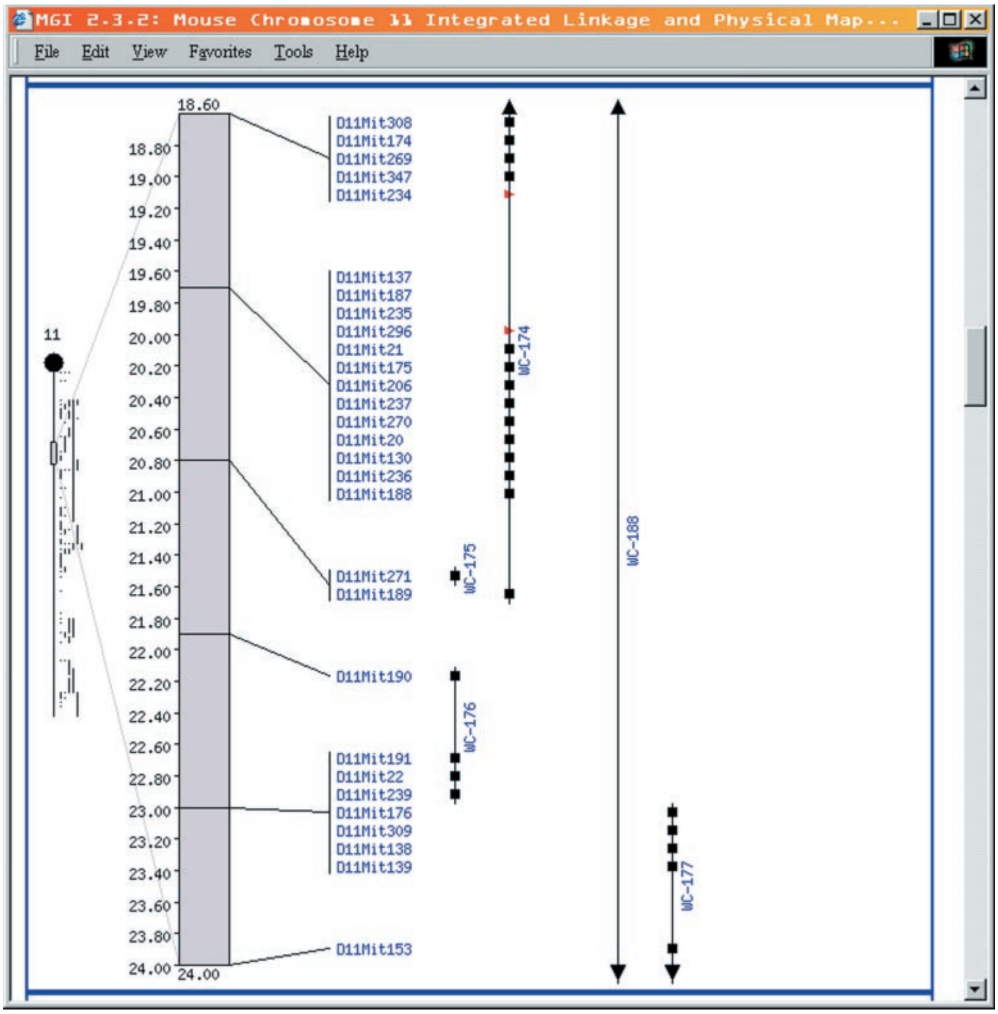

**Figure 4.** A Whitehead Institute/MIT CGR integrated linkage and physical map of chromosome 11 from 18.6–24 cM. The black boxes indicate regions where the physical and genetic maps are in agreement, the red triangles denote regions where the two maps are not in agreement this gene from every category in one search (Figure 6).

### Chromosome Committee reports

This link goes directly to the same chromosome committee reports that can be reached by following the 'Genetic and Phenotypic Data' link from the main page.

### Database reports

This links directly to the same reports as the database reports link in the Genetic and Phenotypic Data section.

### Contributed data sets

This is a collection of data contributed to MGD by external authors that has not been incorporated into the database. There are Excel tables and text files as supplied by the authors and a link to the submission guidelines for those who wish to contribute data.

### Prototypes

This section is a collection of tools or data, the presentation of which is not yet finalized.

Copyright © 2000 John Wiley & Sons, Ltd.

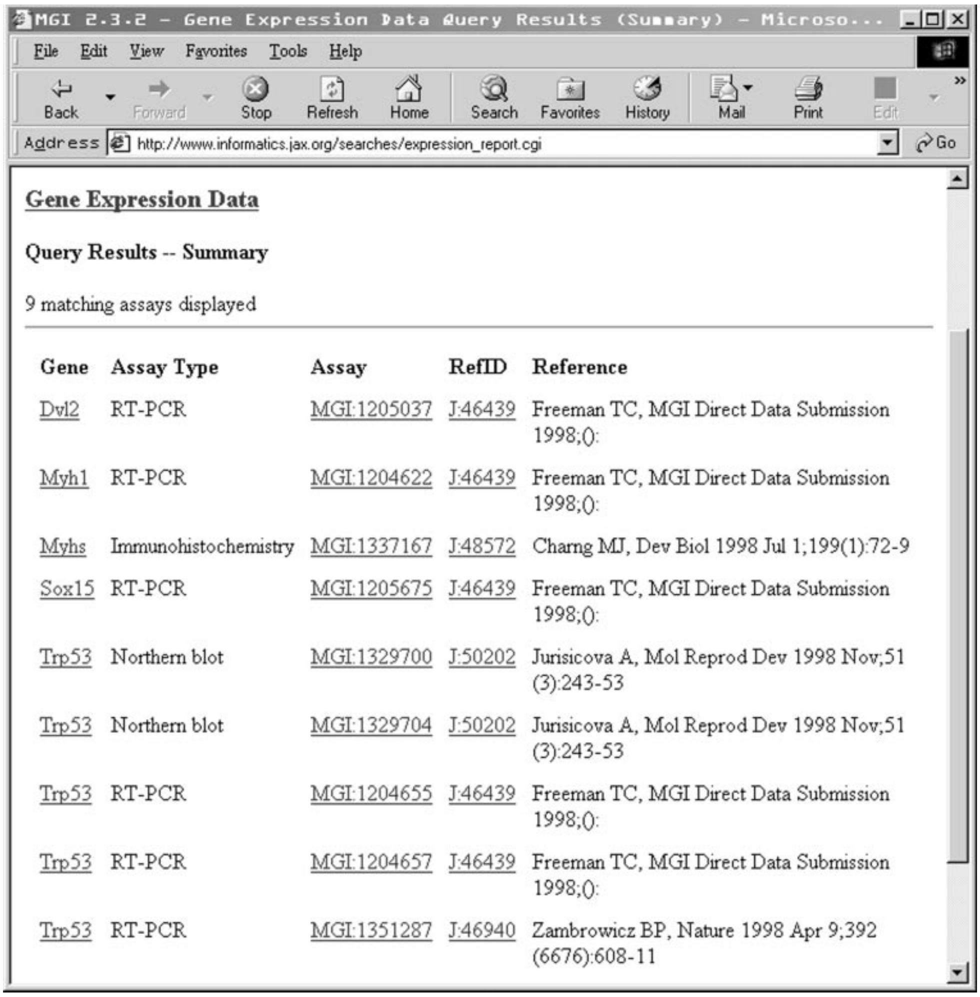

Gene Expression Data

Query Results -- Summary

9 matching assays displayed

| Gene | Assay Type | Assay | RefID | Reference |
|---|---|---|---|---|
| Dvl2 | RT-PCR | MGI:1205037 | J:46439 | Freeman TC, MGI Direct Data Submission 1998;0: |
| Myh1 | RT-PCR | MGI:1204622 | J:46439 | Freeman TC, MGI Direct Data Submission 1998;0: |
| Myhs | Immunohistochemistry | MGI:1337167 | J:48572 | Charng MJ, Dev Biol 1998 Jul 1;199(1):72-9 |
| Sox15 | RT-PCR | MGI:1205675 | J:46439 | Freeman TC, MGI Direct Data Submission 1998;0: |
| Trp53 | Northern blot | MGI:1329700 | J:50202 | Jurisicova A, Mol Reprod Dev 1998 Nov;51 (3):243-53 |
| Trp53 | Northern blot | MGI:1329704 | J:50202 | Jurisicova A, Mol Reprod Dev 1998 Nov;51 (3):243-53 |
| Trp53 | RT-PCR | MGI:1204655 | J:46439 | Freeman TC, MGI Direct Data Submission 1998;0: |
| Trp53 | RT-PCR | MGI:1204657 | J:46439 | Freeman TC, MGI Direct Data Submission 1998;0: |
| Trp53 | RT-PCR | MGI:1351287 | J:46940 | Zambrowicz BP, Nature 1998 Apr 9;392 (6676):608-11 |

**Figure 5.** The results of a gene expression data search for chromosome 11 from 35–39 cM, with no specified developmental stage or assay type. Each gene links to detailed information on the gene, each assay links to the GXD data, and each 'RefID' links to the reference in question

### Mouse BLAST

This prototype is a Washington University BLAST, Version 2 (WU-BLAST 2.0) search of the mouse and rodent sequence database. Clicking on 'Mouse BLAST' at the top of the page takes you to a well-structured Help page with instructions on how to use the search and examples of searches. There are also links to more detailed information on topics such as options for repeat masking and the databases available. Users can search using sequence or by giving an MGI Accession No. as the source for the query sequence. It should be noted, though, that the search does seem to run quite slowly.

### Mouse tumor biology (MTB) database

The MTB database contains an enormous amount of data on mouse tumours, including links to relevant references and some images of tumour pathology. The main page offers a quick search of tumour type by organ or tissue of origin. There are also more advanced searches with search fields such as tumour-related gene/loci, transgene, tumour type, incidence and pathology, and literature citation. A typical search result (Figure 7) is a table of tumour records, with links to more detailed information, on the genes mutated (or expressed as transgenes) in that tumour and on the strain itself. Clicking

Copyright © 2000 John Wiley & Sons, Ltd.

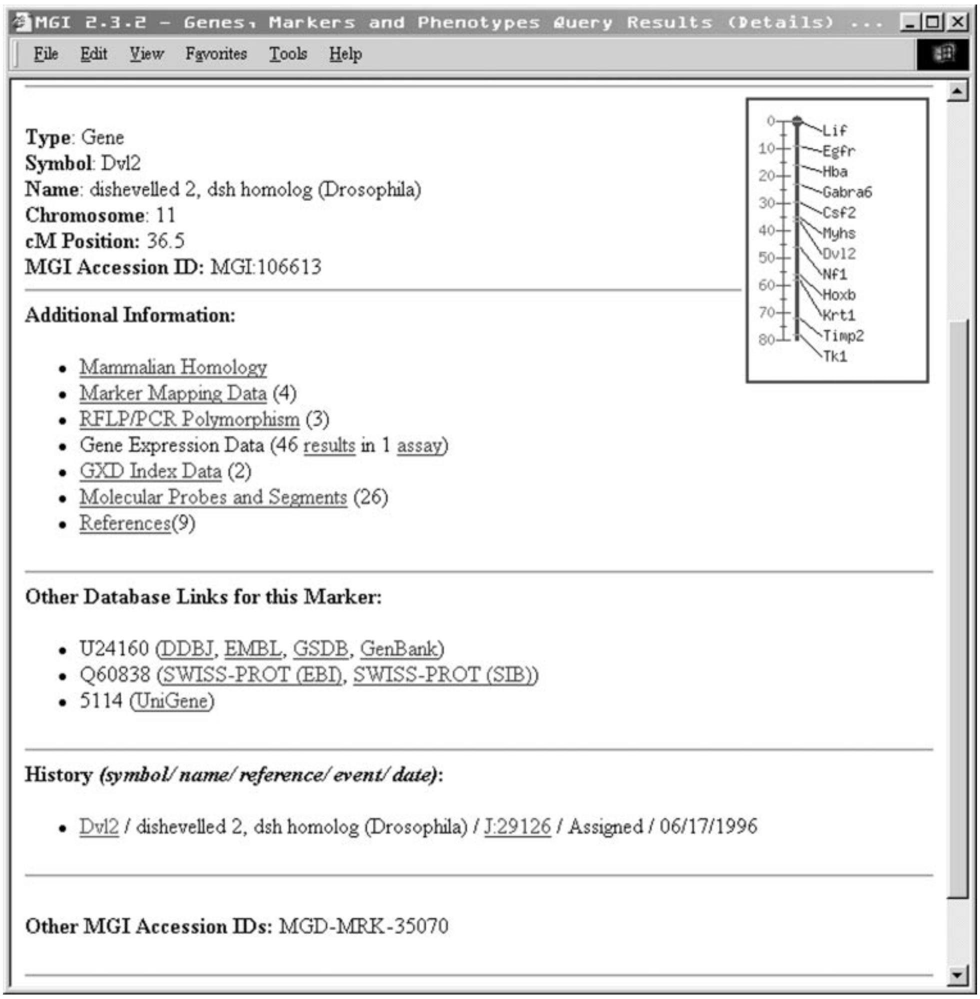

**Figure 6.** The result of an Accession number search for dvl2 accession number: MGI: 106613. This search will find any MGI records pertaining to dvl2. The results are grouped by category. Links to entries for this gene in other databases are also provided through to a strain entry leads to a table with links to information on the pathology and incidence of the tumour or tumours in that strain, on the reference in which it was described and on the strain background used. This database will surely be of great interest to researchers working on mammalian cancer biology. Non-mouse researchers would be best advised to use the mammalian homology search to ascertain the gene symbol of the mouse orthologue of a gene of interest, before searching with that term in MTB. Links to counts of tumour records and information on the controlled vocabularies used in mouse tumour biology are also provided from the main page of MTB.

### Rat genome data

It should be noted that these pages only contain data from the Whitehead Institute/MIT center and the Medical College of Wisconsin rat projects. Searches of genetic markers, polymorphisms and maps are provided. There are links to the sections of MGD that contain rat data; the Mammalian Homology section and the data on inbred strains of mice and rats. The site authors are considering providing a rat database, to assess the needs of users; there is a rat data survey which users can complete. There are also links to the MGI user support pages and the e-mail list service page, since there is a rat-specific e-mail bulletin service.

Copyright © 2000 John Wiley & Sons, Ltd.

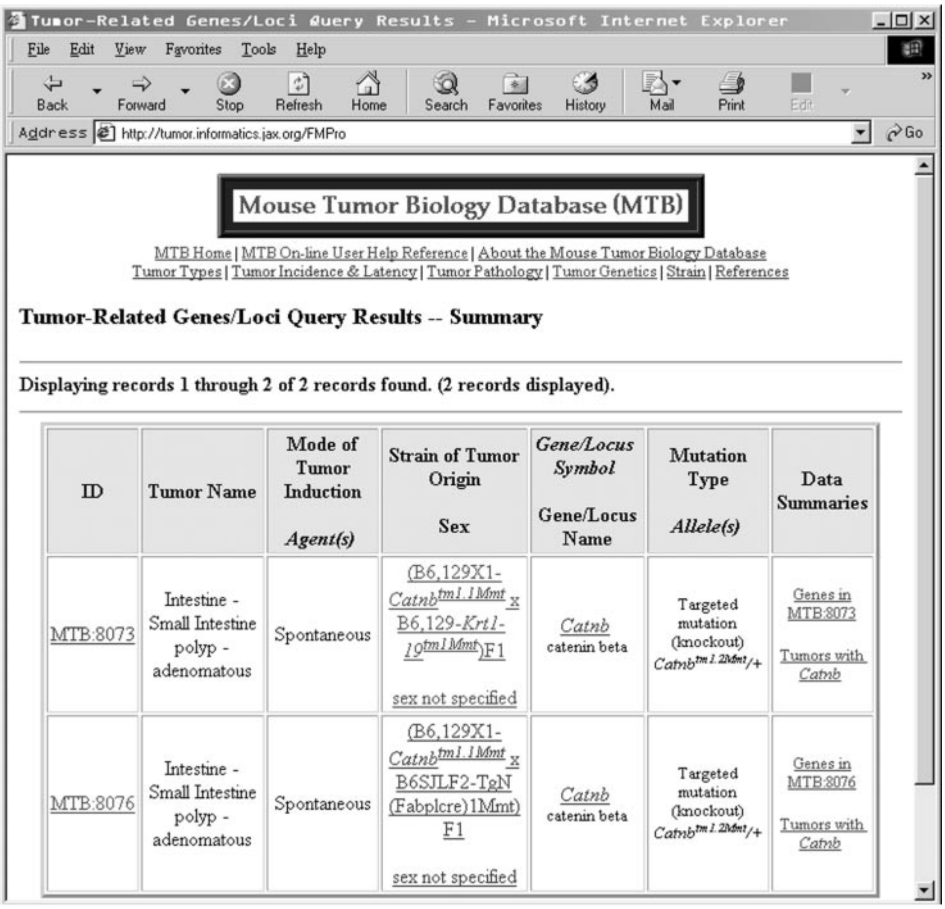

**Figure 7.** The result of a tumour-related gene/loci search using gene name 'contains' with the query term 'catenin'. β-Catenin is a critical mediator of the Wnt signal transduction pathway involved in colorectal cancer. The table has basic information about each tumour, and has links through to more detailed entries on tumour pathology, strain background and on the gene used as the query term

### Other MGI resources

### Data and nomenclature submissions

This page has a link to instructions on how to submit data to MGD. There is also a link to information on nomenclature and the locus symbol registry. The final link is for chromosome committee members to use to submit or update reports.

### User support

This links to information on how to register as a user and how to contact the user support service. The express mail form can also be used to send in comments and suggestions in addition to requesting help.

### MGI e-mail list service

The MGI run a mouse and a rat e-mail list service. The list acts as a forum for discussion of research topics of common interest. Users can visit the forum as a guest before deciding to subscribe.

### Tools for developers

This section is mainly intended for authors of other databases. It provides information on how to make links from your database to the MGI resources, information on the database schema and on how to download material from their FTP server.

Copyright © 2000 John Wiley & Sons, Ltd. 

### Summary

This is an excellent website, with a wide range of well-supported searches and an impressive selection of tools. There is clearly an enormous amount of data in the databases behind these pages, and the topic-related breakdown of the searches makes it much easier to get hold of the information required.

There are several parts of the database that will be of interest to those working on other mammalian genomes, in particular the homology search tool, the Oxford grids and the comparative map-drawing tool. It would be nice to see the incorporation of the mouse sequence data into the comparative map tool. This could be done by including links to regions of sequence comparison, for example by using vertical lines alongside the map which indicate the locations of sequenced contigs.

The expression database tools are most impressive. There is a high degree of interlinking between the references and the data, and the availability of raw data is excellent. The Mouse Tumour Biology database will be of interest to those working on cancer in any mammalian system; the mouse is a tremendously important model for this disease and an enormous amount of data is made available in a well-organized system. The Help pages reached by clicking on the titles of some of the pages could perhaps be improved by giving examples of query terms for every field of the form in question.

### Equipment details

This review was completed using a Dell PIII Inspiron 3700 laptop, running Windows 98, with a permanent 10 Mbps Ethernet/Internet connection and a screen with $1024 \times 768$ pixels resolution. The primary software used was Internet Explorer Version 5.

### MGI mirror sites

UK
**http://mgd.hgmp.mrc.ac.uk**
At the UK MRC Human Genome Mapping Project Resource Centre.

Japan
**http://mgd.niai.affrc.go.jp**
At the National Institute of Animal Industry/Japan Animal Genome Database.

Australia
**http://mgd.wehi.edu.au/mgd**
At the Walter and Eliza Hall Institute of Medical Research, Melbourne, Australia.

### Related sites

Whitehead Institute for Biomedical Research/MIT Center for Genome Research
**http://www-genome.wi.mit.edu/**

Links to rodent genome databases at MRC HGMP Genome Web
**http://www.hgmp.mrc.ac.uk/GenomeWeb/rodent-gen-db.html**

The Mouse Genome Project at Baylor College of Medicine
**http://www.mouse-genome.bcm.tmc.edu/**

The MRC Mammalian Genetics Unit
**http://www.mgu.har.mrc.ac.uk/**

'Mouse Sequences in SWISS-PROT' has information about mouse entries in SWISS-PROT and links to mouse databases
**http://www.ebi.ac.uk/**~magrane/

The Whole Mouse Catalog at Muridae.com
The Mouse Atlas and Gene Expression Database Project
**http://genex.hgu.mrc.ac.uk/**

RATMAP at the Lundberg Laboratory, Göteborg University
**http://ratmap.gen.gu.se/**

### References

Blake JA, Richardson JE, Davisson MT, Eppig JT, the Mouse Genome Database Group. 1999. The Mouse Genome Database (MGD): genetic and genomic information about the laboratory mouse. *Nucleic Acids Res* **27**: 95–98.

Bult CJ, Krupke DM, Sundberg JP, Eppig JT. 2000. Mouse Tumor Biology Database (MTB): enhancements and current status. *Nucleic Acids Res* **28**: 112–114.

Ringwald M, Mangan ME, Eppig JT, Kadin JA, Richardson JE, the Gene Expression Database Group. 1999. GXD: a gene expression database for the laboratory mouse. *Nucleic Acids Res* **27**: 106–112.

Copyright © 2000 John Wiley & Sons, Ltd.

Some of the sites reviewed will already be known to you but perhaps their content will be less well-known. The Website Review is intended to help you discover new sites of interest, but also to provide a rapid and convenient means of revealing what you always knew was there but never had the time or inclination to look at. These articles are a personal critical analysis of the Website. If you have any information about sites you think are worthy of being more widely known, the Managing Editor would be pleased to hear from you.

This Website Review was written by Dr Joanne Wixon (Managing Editor) with the assistance of Mark Strivens (Head of Informatics, MRC Mammalian Genetics Unit, Harwell, UK). Thanks also to Dr Mark Albertella (Cancer and Infection Research, AstraZeneca, Alderley Park, UK) for reviewing the Mouse Tumour Biology database.

*The* **Genomics** *Website at Wiley*

**www.wiley.co.uk/genomics**

The **Genomics** website at Wiley is a new and **DYNAMIC** resource for the genomics community, offering **FREE** special feature articles and new information **EACH MONTH**.

Find out more about *Comparative and Functional Genomics*, and how to view all articles published this year **FREE OF CHARGE**!

Visit the **Library** for hot books in Genomics, Bioinformatics, Molecular Genetics and more.

Click on **Primary Research** for information on all our up-to-the minute journals, including: *Genesis*, *Bioessays, Gene Function and Disease*, and the *Journal of Gene Medicine*.

Let the **Genomics** website at Wiley be your **guide** to genomics-related web sites, manufacturers and suppliers, and a calendar of conferences.

WILEY

1516

Copyright © 2000 John Wiley & Sons, Ltd. 
